# Traditional Chinese decoction Si Zhi Wan attenuates ovariectomy (OVX)-induced bone loss by inhibiting osteoclastogenesis and promoting apoptosis of mature osteoclasts

**DOI:** 10.3389/fphar.2022.983884

**Published:** 2022-09-13

**Authors:** Qingman He, Kanghua Fu, Huan Yao, Shujun Wei, Li Xiang, Sixian Liu, Tao Chen, Yongxiang Gao

**Affiliations:** ^1^ Affiliated Hospital of Chengdu University of Traditional Chinese Medicine, Chengdu, China; ^2^ Sichuan Police College, Luzhou, China; ^3^ International Education College, Chengdu University of Traditional Chinese Medicine, Chengdu, China

**Keywords:** Si Zhi Wan, traditional Chinese decoction, osteoclastogenesis, apoptosis, NF-κB signaling pathway

## Abstract

Si Zhi Wan (SZW) is a traditional Chinese decoction used for osteoporosis treatment. Currently, the effect of SZW on ovariectomy (OVX)-induced bone loss and the underlying mechanisms remain unknown. Herein, we investigated the therapeutic effect of SZW on osteoporosis and explored the underlying mechanisms *in vitro* and *in vivo*. An OVX-induced bone loss model was established *in vivo*. After administration of SZW for 8 weeks, rats were sacrificed, and the uterus was weighted to calculate its index. The femur change was pathologically evaluated using hematoxylin and eosin (H&E) staining. The mineral density of the femur was observed by micro-CT. RAW264.7 cells were activated by receptor activator of nuclear factor-κB ligand (RANKL) *in vitro*. The effect of SZW on osteoclastogenesis was evaluated using tartrate-resistant acid phosphatase (TRAP) staining, Western blotting, and RT-PCR. The pro-apoptosis effect of SZW on mature osteoclasts was examined after induction of osteoclast maturation. Finally, the effect of SZW on the NF-κB pathway was evaluated. Our results demonstrated that SZW ameliorated OVX-induced bone loss in rats. In addition, SZW inhibited osteoclastogenesis and attenuated osteoclast-mediated bone resorption *in vitro* and *in vivo*. SZW also promoted apoptosis of mature osteoclasts. Mechanically, SZW exerts its effects by suppressing the NF-κB pathway. Overall, these findings demonstrated that SZW may be a potentially effective alternative treatment for osteoporosis.

## Introduction

Osteoporosis is a common systemic metabolic bone disease characterized by reduced bone mass, deformed microarchitecture, and decreased bone strength, which increases bone fragility and fracture risk (Tu et al., 2018). Globally, there are 200 million osteoporosis cases, of which 9 million develop fractures (Geiker et al., 2020). Although the incidence of osteoporosis varies by geographical region, gender, and age, its prevalence is significantly high in people aged 60 years or older and in women. Over the past few decades, various drugs have been used to prevent and treat osteoporosis. However, their efficacy is unsatisfactory (Tu et al., 2018). Raloxifene is a selective estrogen receptor modulator that increases the risk of blood clots and stroke (Lu et al., 2020). Meanwhile, hormone replacement therapy (HRP) is only recommended for postmenopausal women (Gosset et al., 2021). Even though Bisphosphonates effectively reduces bone loss, their long-term use increases the risk of atypical femoral fractures and jaw osteonecrosis (Ensrud and Crandall, 2021).

Bone homeostasis maintains the balance between bone formation and resorption (Rachner et al., 2011), and an imbalance in these processes is critical for osteoporosis development. Excessive resorption and low bone formation induce osteoporosis (Kaur et al., 2020). In postmenopausal osteoporosis, abnormally high resorption or demineralization is the primary cause of osteoporosis. Osteoclasts are large, multinucleated cells that differentiate from monocyte-macrophage lineage precursor cells and are the main cell type that regulates in bone resorption. Osteoclastosis is regulated through numerous signaling molecules (Udagawa et al., 2021). Osteoclastogenesis and bone resorption require receptor activator of nuclear factor-κB ligand (RANKL) and macrophage-colony stimulating factor (M-CSF). M-CSF is essential for the survival, formation, and function of osteoclasts. RANKL is a transmembrane molecule belonging to the tumour-necrosis-factor cytokine family. RANKL stimulates NF-κB and mitogen-activated protein kinases (MAPKs) signaling pathways by binding to RANK, which then activates downstream transcription factors such as C-FOS and nuclear factor of activated T cells type c1 (NFATc1), molecules central to the regulation of osteoclast differentiation (Boyle et al., 2003; Kodama and Kaito, 2020). Numerous studies have shown that strategies targeting osteoclastogenesis can treat osteoporosis (Boyce, 2013; Kitaura et al., 2020).

Medicinal plants are an important part of the traditional Chinese medicine system in China, and these plants are considered key ingredients for the therapeutic effects of Chinese medicine. Si Zhi Wan (SZW) is a traditional Chinese herbal formula that comprises a mixture of *Eclipta Prostrata L.* (EPL), *Fructus Ligustri Lucidi* (FLL), *Angelicae Sinensis Radix* (ASR), and *Epimedii Folium* (EF), has been clinically used for the treatment of osteoporosis. The composition of the SZW is based on the theory of traditional Chinese medicine and relevant studies. Specifically, Er Zhi Wan (EZW), a classic Chinese formulation, which contains EPL and FLL, has been reported to have beneficial effects on osteoporosis (Cheng et al., 2011; Liu et al., 2014; Chen et al., 2017). Pharmacological studies have shown that ASR (Kong et al., 2014) and EF (Kim et al., 2018) can treat osteoporosis. Therefore, we prepared SZW by adding ASR and EF to EZW. To date, no study has evaluated the therapeutic potential of SZW on osteoporosis. In this study, we explored this phenomenon *in vitro* and *in vivo.*

## Materials and methods

### Reagents

Mouse M-CSF was purchased from HUABIO (Zhejiang, China). Mouse RANKL was purchased from R&D (Minneapolis, MN, United States). The TRAP staining kit was purchased from Sigma-Aldrich (St. Louis, MO, United States), whereas the H&E and Masson trichrome staining kits were purchased from BaSO (Zhuhai, China).

### Preparation and analysis of SZW

The SZW decoction consists of four Chinese medicinal botanical drugs: *Ecliptae Herba* (Asteraceae; *Eclipta Prostrata L.*), *Glossy Privet Fruit* (Oleaceae; *Fructus Ligustri Lucidi*), *Chinese Angelica* (Umbelliferae; *Angelicae Sinensis Radix*), and *Short-horned Epimedium Herb* (Berberidaceae; *Epimedii Folium*). The major identified compounds of above medicinal botanical drugs are wedelolactone, salidroside, ferulic acid, and icariin. All the medicinal botanical drugs were obtained from the Sichuan Provincial Hospital of Traditional Chinese Medicine and used in the ratio of 20:20:10:20 g, respectively, in the order listed. Crude components were authenticated microscopically and macroscopically. All voucher specimens were deposited at the Research Center for Inheritance and Transformation of Classic Famous Formulas, Chengdu University of Traditional Chinese Medicine.

To prepare the lyophilized powder of SZW extracted liquid, all botanical drugs were soaked in 10 times purified water (1:10, w/v) for 30 min. Then, the filtrates were concentrated in vacuum under 60 °C with a rotary evaporator (N1200-BV, Tokyo Rikakikai Co. Ltd., Tokyo, Japan), and followed freeze-dried (LGJ-12B freeze-drier, Shanghai GIPP Co. Ltd, Shanghai, China) to prepare the SZW water extracts. Then, the SZW extracts were powdered and stored in a dryer for future use. The quality of SZW was analyzed using High-performance liquid chromatography (Supplementary Figure S1).

### Cell culture

RAW264.7 cells were purchased from Procell Life Science and Technology Co., Ltd. (Wuhan, China) and were cultured in Dulbecco’s Modified Eagle Medium (DMEM; Gibco, NY, United States) supplemented with a mixture of 1% penicillin/streptomycin and 10% fetal bovine serum (FBS) at 37°C in 5% CO_2_ humidified air.

### Extraction of bone marrow precursors and *in vitro* osteoclastogenesis

Osteoclastogenesis was induced as previously described (Chevalier et al., 2021). Briefly, bone marrow cells extracted from the femurs and tibiotarsus of 4 - 6-week-old C57BL/6 male mice were incubated at 37°C for 24 h under 5% CO_2_ in 10-cm Petri dishes in α-MEM supplemented with 10% FBS and 1% penicillin/streptomycin. Nonadherent cells were collected and cultured in α-MEM supplemented with 40 ng/mL M-CSF for 3 days. The adherent cells were used as bone-marrow-derived monocytes/macrophages (BMMs). BMMs were digested using trypsin and resuspended, then seeded in 6, 24, or 96-well plates at a density of 5 × 10^5^ cells/mL in α-MEM supplemented with 40 ng/mL M-CSF and 100 ng/ml RANKL. The culture medium was replaced daily until mature multinuclear osteoclasts were formed.

### Cell viability assay

The cytotoxic effects of SZW on RAW264.7 cells were evaluated using the Cell Counting Kit (CCK, Solarbio, Beijing, China)-8 assay. Briefly, the cells were seeded in 96-well plates at a density of 5×10^3^ cells/well in 96-well plates and incubated for 24 h. The cells were treated with vehicle (ddH_2_O) and different SZW concentrations (5, 10, 25, 50, 100, 250, 500, 1000 μg/ml). Fresh media supplemented with CCK-8 reagent was added after 24 h, followed by further incubation at 37 °C for 1 h. The absorbance for each well was read at 450 nm using a microplate reader (Molecular Device, Shanghai).

### Toluidine blue staining of bovine bone slices

Cells were seeded onto the slices at a density of 1 × 10^5^ cells/well with the indicated treatment. The medium was replaced every day. After 10 days of culture, the slices were washed with PBS followed by fixation with 2.5% glutaraldehyde for 10 min. Cells were then dislodged from bone slices via ultrasonication in 0.25 M NH_4_OH. Finally, slices were stained by 0.1% toluidine blue (Solarbio, Beijing, China) for 10 min at room temperature and rinsed with distilled water 3 times to excluderesidues. The resorption pits are stained in dark blue and images were taken via light microscopy.

### Flow cytometry

Flow cytometry was performed to determine the apoptosis rate of osteoclasts. Briefly, osteoclasts were harvested and stained with Annexin V-FITC and propidium iodide (PI) (Beyotime) for 40 min at room temperature. The osteoclasts were then washed twice with PBS, and the fluorescence data obtained from the cell population were analyzed with the CellQuest software (BD Biosciences, Franklin Lakes, NJ, United States).

### Animals

Female 8-week-old Sprague Dawley rats (200 ± 15 g) were purchased from SPF Lab Animals Technology Co., Ltd. (Beijing, China) and allowed to acclimatize for 1 week before the experiments. Experiments were performed in compliance with the National Institutes of Health Guidelines for Care and Use of Laboratory Animals. The protocol for this study was approved by the Bioethics Committee of Chengdu University of Traditional Chinese Medicine.

### Ovariectomy (OVX)-induced osteoporosis rat model

The OVX-induced osteoporosis rat model was established as previously described (Yousefzadeh et al., 2020). Briefly, the mice were starved for 8 h and injected intraperitoneally with 3% sodium pentobarbital (40 mg/kg) for anesthetization. Longitudinal incisions of approximately 1 cm were made on both sides of the rats’ back (1 cm below the rib cage, about 3 cm from the spine). A small adipose tissue around the ovaries of mice in the Sham group was extracted. OVX was induced in the remaining rats by removing both ovaries. All rats were injected with penicillin-sodium intramuscularly for 3 days to prevent infection. On day seven post-operation, OVX rats received 1 mg/kg of dexamethasone sodium phosphate twice a week, while mice in the Sham group received saline water daily for 8 weeks through intramuscular injection. OVX rats were separated randomly into four groups of OVX with vehicle (OVX, n = 6), OVX and low SZW dose (low dose, n = 6), OVX and medium SZW dose (medium dose, n = 6), and OVX and high dose SZW (high dose, n = 6). Rats in the low-, medium-, and high-dose SZW groups should receive 4, 8, and 16 g/kg SZW (crude botanical drugs) everyday, respectively. Because the extraction rate of SZW lyophilized powder from the crude botanical drugs is 10%, the doses of SZW lyophilized powder extract administered to rats in the low-, middle- and high-dose groups were 0.4, 0.8, and 1.6 g/kg, respectively. Mice in the Sham and the vehicle group received 1 ml saline/100 g of body weight through intragastric injection 6 times a week for 8 weeks.

### Organ coefficients

The uterus and vagina of the rats were removed and weighed before and after drying at 65°C. Their corresponding organ coefficients were calculated as follows: organ coefficient = organ weight/body weight × 100%.

### Micro-CT imaging analysis

Three rats from each experimental group were randomly selected. The right distal femur of rat was excised, fixed in 4% paraformaldehyde for 48 h, and rinsed under running water for 3 h. Micro-CT was performed using the SkyScan 1176 high-resolution micro-computed tomography scanner (SkyScan, Knotich, Belgium). The scanning parameters were 9 μm per layer, 80 kV (voltage) and 100 mA (current). The 3D-images were obtained and the parameters including bone mineral density (BMD), bone volume/total volume (BV/TV), trabecular number (Tb. N), trabecular separation (Tb. Sp), trabecular thickness (Tb. Th), and connectivity density (Conn. D) were assessed using the CTAn software (Bruker micro-CT, Kontich, Belgium).

### Histological analysis

The right distal femur of rat from Micro-CT analysis were rinsed three times in tap water for 1 h and 20 min in distilled water. The femurs were decalcified at 37°C for 2 weeks, dehydrated in gradient alcohol, embedded in paraffin, and cut into 5 μm thick sections as previously described (Yao et al., 2022). The samples were stained with H&E, Masson trichrome, and TRAP staining. For trabeculae area measurement, the ImageJ software was used to open the pathological picture and circle the trabecular bone part. After that, add the trabecular bone area of each part, and then convert according to the picture and the actual ratio to obtain the actual trabecular bone area.

### Western blotting

Proteins in the bone tissue and cells were extracted using radioimmunoprecipitation assay buffer (RIPA) and quantified using a Bicinchoninic Acid Protein Assay (BCA) Kit (Beyotime, Jiangsu, China). The proteins were separated using 10% sodium-dodecyl-sulfate polyacrylamide gel electrophoresis and transferred onto polyvinylidene difluoride membranes. The membranes were blocked with 5% skimmed milk in Tris-buffered saline for 1 h, incubated with primary antibodies overnight at 4 °C, washed with Tris-buffered saline Tween-20, and incubated with horseradish peroxidase-conjugated secondary antibody for 1 h at room temperature. The primary antibodies used in this study are listed in Supplementary Table S1.

### Quantitative real-time PCR

Total RNA was extracted from bone tissues and cells using TRIzol™ Reagent (Invitrogen) according to the manufacturer’s instructions. Quantitative reverse transcription-polymerase chain reaction (qRT-PCR) of the RNA was performed as described previously (Yao et al., 2022), using the iScript™ cDNA Synthesis Kit (Yeasen, Shanghai, China). mRNA levels were analyzed using qRT-PCR (Bio-Rad) using Hieff Unicon^®^ universal Blue qPCR SYBR Green Master Mix (Yeasen). The total PCR volume was 20 μL. The primers used in the present study are listed in Supplementary Table S1.

### Measurement of cross-linked C-telopeptide of type Ⅰ collagen (CTX I)

Rat serum CTX I levels were determined using a Rat CTX I ELISA Kit (Elabscience, Wuhan, China) according to the manufacturer’s instructions.

### Statistics

Quantitative data are presented as means ± standard error of mean (SEM). Comparisons of multiple groups were performed using one-way analysis of variance with Tukey’s post hoc test. For the time-dependent data or two independent variables, two-way ANOVA was used. All the statistical data were analyzed in SPSS 21.0 (SPSS Inc., Chicago). *p*-values < 0.05 were considered indicative of statistical significance.

## Results

### SZW ameliorated ovariectomy-induced bone loss in rats

**FIGURE 1:**
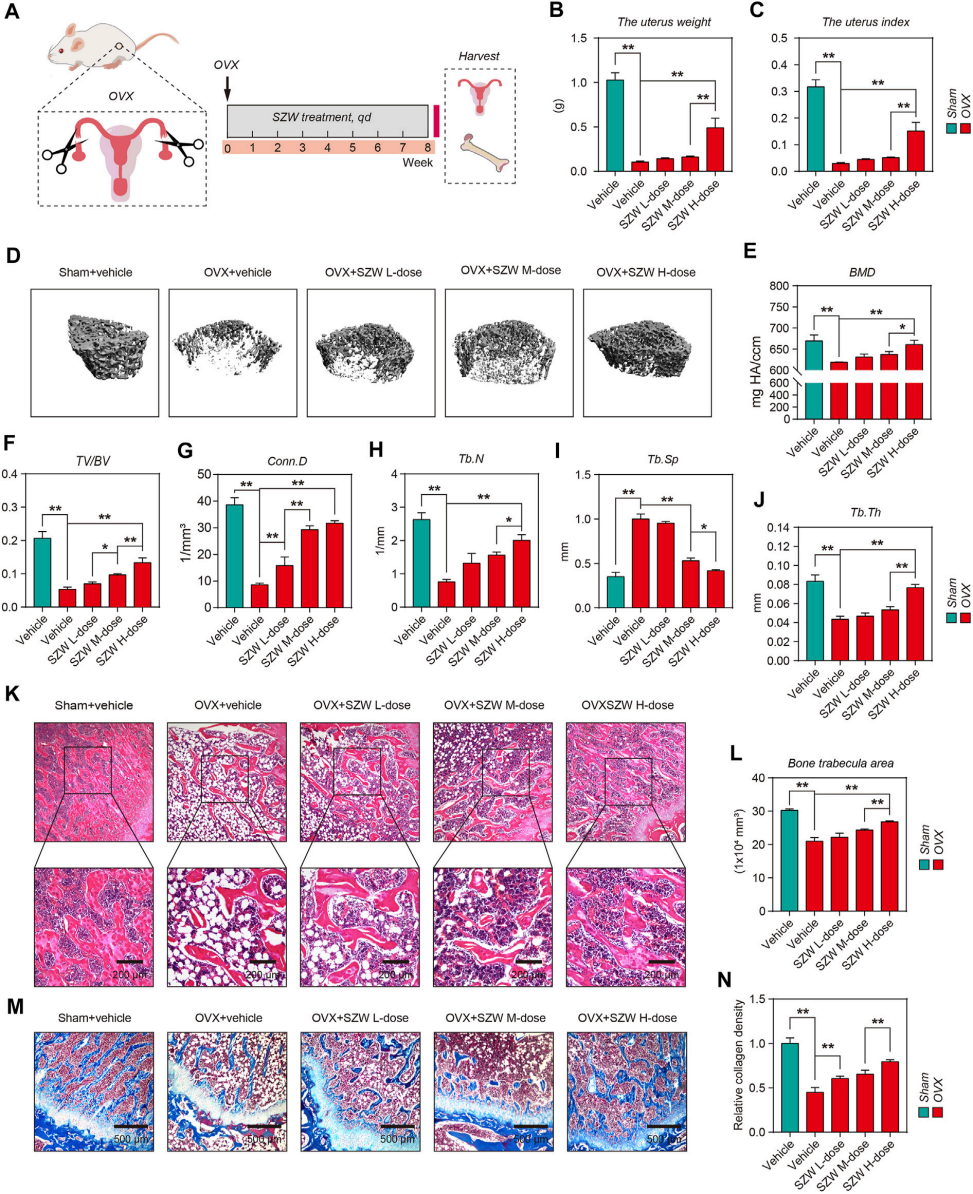
SZW ameliorated ovariectomy-induced bone loss in rats **(A)** Schematic diagram of the *in vivo* experiments **(B)** The weight of uterus in each group **(C)** The uterus index of rats in each group **(D)** Representative μCT images of femur bone **(E–J)** Quantitative analyses of parameters of bone microstructure and cortical bone, including BMD, BV/TV, Conn. D, Tb.N, Tb. Sp, and Tb.Th **(K)** Representative H&E staining of femur sections in different groups after OVX **(L)** The quantitative analysis of bone trabecula area **(M)** Representative Masson’s trichrome staining of the femur sections **(N)** Relative collagen density was quantified using ImageJ. Data are presented as the mean ± SEM (n = 6). *p < 0.05, **p < 0.01. BMD, bone mineral density; BV/TV, bone volume per tissue volume; Conn. D, connectivity density; Tb.N, trabecular number; Tb. Sp, trabecular separation; Tb.Th, trabecular thickness; ns, non-significant.

The pharmacodynamic effect of SZW on osteoporosis was evaluated using an OVX rat model. On day two post-operation, the rats were treated with different doses of SZW (0.4, 0.8, and 1.6 g/kg) and vehicle (water) daily for 8 weeks (Figure 1A). The uterus and femur bones of the rats were extracted after the treatment for further analyses. The uterine weight was lower for mice in the OVX group than those in the sham group (Figure 1B). Uterine weight and index were highest for rats administered with 1.6 g/kg SZW (Figure 1C). The structural features of the right distal femurs were evaluated using the 3D-μCT. The results (Figures 1E–J) showed that loss of bone mass was significantly higher in the OVX group than in the SZW treatment group. SZW reversed the bone abnormalities, including BMD, BV/TV, Conn. D, Tb.N, Tb. Sp, and Tb.Th, caused by OVX (Figures 1E– J). Further histopathological analysis revealed that OVX decreased bone trabecula area of the right distal femur, but SZW treatment prevented this phenomenon (Figures 1K,L). Masson trichrome staining and the quantitative collagen density results showed that SZW enhanced collagen expression in a dose-dependent manner (Fig. 1M, N). In addition, serum biochemical assay showed that SZW (1.6 g/kg) had no effect on liver injury-related indicators including aspartate aminotransferase (AST) and alanine aminotransferase (ALT), indicating that SZW had no liver toxicity (Supplementary Figure S2). Overall, these findings demonstrated that SZW improved OVX-induced bone loss in rats.

### SZW inhibited osteoclastogenesis *in vitro* and *in vivo*

**FIGURE 2:**
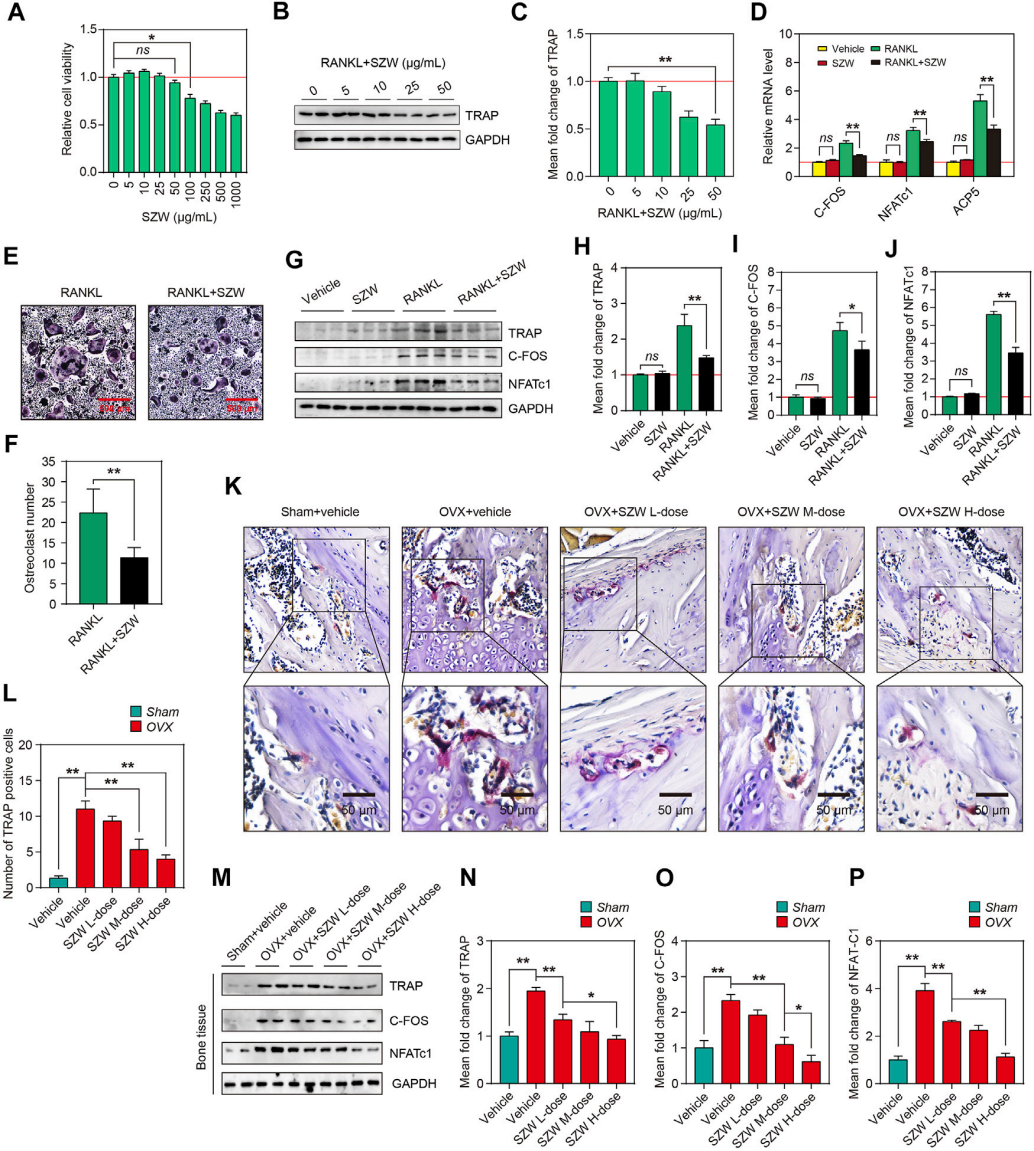
SZW inhibited osteoclastogenesis *in vitro* and *in vivo*. RAW264.7 cells were treated with different concentrations of SZW (0, 5, 10, 25, 50, 100, 250, 500 and 1000 μg/ml) for 24 h **(A)** The viability of RAW264.7 cells was determined by the CCK-8 assay. Values of treatment groups were normalized to the control group (representing 100% cell viability). Data are expressed as the mean ± SEM (n = 6) **(B)** Western blotting analysis showing the changes in TRAP protein levels in RAW264.7 cells **(C)** The mean fold change of TRAP expression level was quantified using ImageJ **(D)** RT-PCR analysis of the mRNA levels of osteoclastogenesis-related genes, NFATc1, ACP5, and C-FOS, in the control RAW264.7 cell and RAW264.7 cell treated with RANKL and RANKL + SZW. Data are expressed as the mean ± SEM (n = 3) **(E)** Representative images of TRAP staining of Raw 264.7 cells **(F)** The number of osteoclasts in the indicated groups **(G)** Representative western blotting images showing TRAP, C-FOS, and NFATc1 expression in RAW264.7 cells **(H–J)** The mean fold changes of TRAP, C-FOS, and NFATc1 expression levels were quantified using ImageJ **(K)** Representative TRAP staining images of femur sections **(L)** Relative TRAP intensity was quantified using ImageJ **(M)** Representative western blotting images showing TRAP, C-FOS, and NFATc1 protein levels in femur tissues of rats **(N–P)** The mean fold changes of TRAP, C-FOS, and NFATc1 protein levels were quantified using ImageJ. Data are expressed as the mean ± SEM (n = 3). ∗*p* < 0.05, ∗∗*p* < 0.01.

Because osteoclasts are responsible for bone resorption in bone remodeling and are key effector cells leading to osteoporosis, and SZW has a therapeutic effect on osteoporosis, we further clarified the effect of SZW on osteoclastogenesis. The effect of SZW on RANKL-induced osteoclastogenesis was evaluated using the monocyte-macrophage RAW264.7 cell line. The optimal SZW concentration was determined using the CCK-8 assay (Figure 2A). A 0–50 μg/ml SZW concentration had no cytotoxicity on RAW264.7 cells. Thus, serial SZW concentrations (0, 5, 10, 25, and 50 μg/ml) were used for the osteoclastogenesis experiment. Western blotting revealed that 50 μg/ml of SZW inhibited the expression of TRAP, an osteoclast-marker, induced by 100 ng/ml RANKL. Thus, 50 μg/ml of SZW was used in the subsequent experiments. RT-PCR assay showed that SZW had no effect on mRNA transcription for osteoclastogenesis-related genes, including C-FOS, NFATc1, and ACP5. However, SZW, in combination with RANKL, inhibited the transcription of these genes. TRAP staining of RAW264.7 cells indicated that SZW inhibited RANKL-induced osteoclastogenesis, indicated by a decrease in giant and multinucleated TRAP-positive osteoclasts (Figures 2E,F). Similarly, SZW reversed RANKL-induced overexpression of TRAP, C-FOS, and NFATc1 proteins but had no effect on the expression of these proteins without RANKL stimulation. TRAP staining on femur sections further demonstrated that OVX increased the number of TRAP-positive osteoclasts in the bone marrow of femur bones, but SZW treatment inhibited this phenomenon (Figure 2K, L). Similarly, Western blotting analysis illustrated that SZW inhibited OVX-induced increase in TRAP, C-FOS, and NFATc1 expression in the rat femoral tissue in a dose-dependent manner (Figures 2M–P). These results demonstrated that SZW prevented OVX-induced bone loss by inhibiting osteoclastogenesis.

### SZW attenuated osteoclast-mediated bone resorption

**FIGURE 3:**
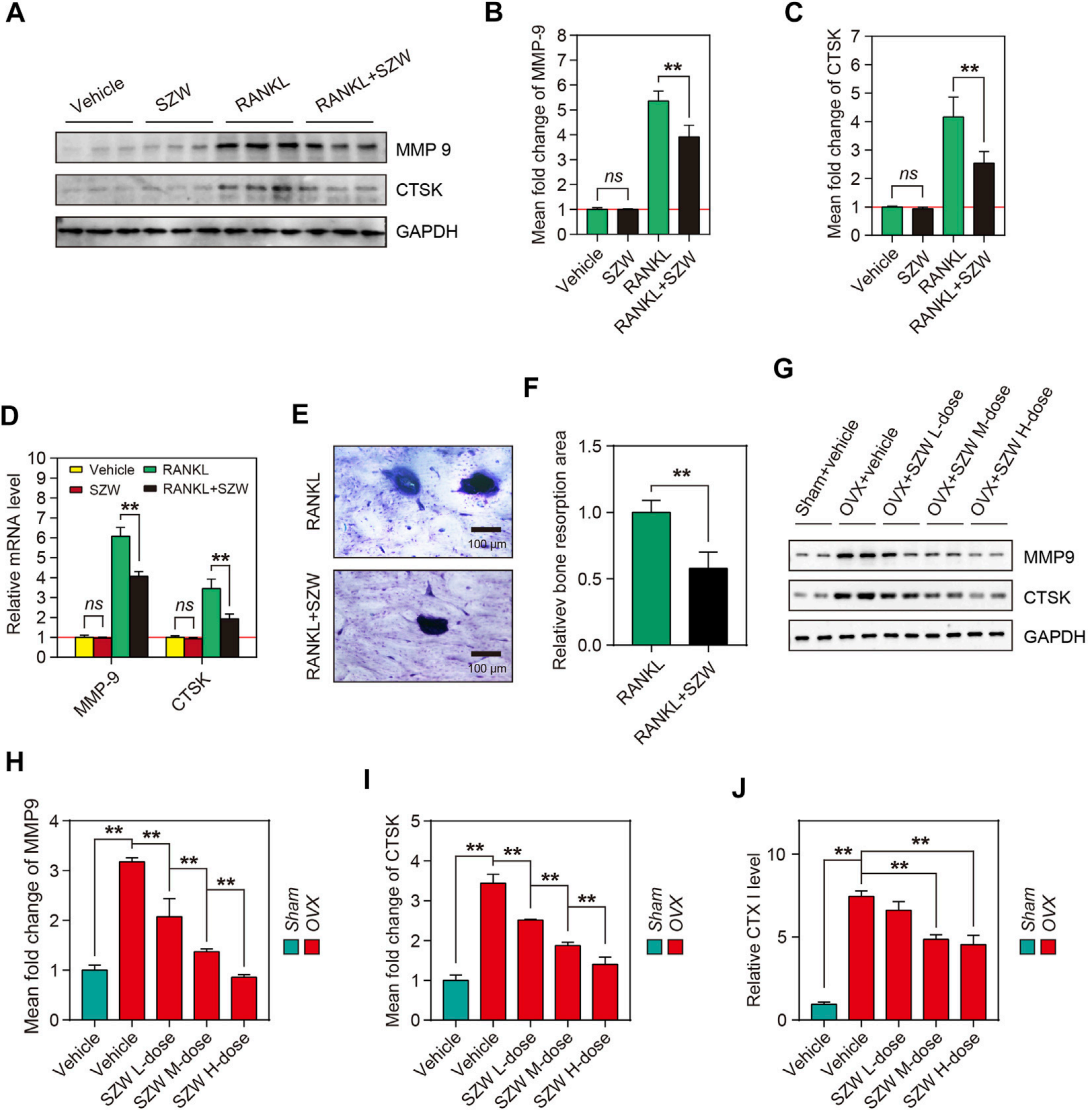
SZW attenuated osteoclast-mediated bone resorption. RAW264.7 cells were treated with vehicle (H_2_O) or SZW in the presence or absence of RANKL **(A)** Representative western blotting images showing MMP9 and CTSK expression in RAW264.7 cells **(B–C)** The mean fold changes of MMP9 and CTSK expression were quantified using ImageJ **(D)** Quantitative real time RT-PCR results showing the expression level of bone erosion-related genes in RAW264.7 cells following vehicle or SZW treatment **(E)** Representative toluidine blue staining of bovine bone slices for osteoclast resorption **(F)** Quantitative analysis of bone resorption pits using ImageJ **(G)** Representative western blotting images showing MMP9 and CTSK expression levels in femur tissue of rats **(H–I)** The mean fold changes of MMP9 and CTSK expression in femur tissues as determined with using ImageJ. Data are expressed as the mean ± SEM (n = 3) **(J)** Rat serum CTX I level was measured using an Elisa kit. Data are expressed as the mean ± SEM (n = 6). ∗*p* < 0.05, ∗∗*p* < 0.01.

Mature osteoclasts resorb bone matrix by secreting acids, proteases (e.g., CTSK), and matrix metalloproteinases (MMPs). Because SZW inhibited osteoclastogenesis *in vitro* and *in vivo*, we hypothesized that SZW could block the bone resorption function of osteoclast. Western blotting and RT-PCR were performed to investigate the effect of SZW on the expression and translation of MMP9 and CTSK genes. The results showed that compared with the RANKL alone, a combination of RANKL and SZW repressed the transcription of mRNA and subsequent expression of MMP9 and CTSK proteins (Figures 3A–D). Additionally, the effects of SZW on *in vitro* bone resorption were determined. In comparison with the RANKL group, the area of bone resorption pits was markedly decreased by SZW treatment (Figures 3E,F). *In vivo* studies further revealed that SZW also inhibited the expression of MMP9 and CTSK in a dose-dependent manner (Figures 3G–I). The serum CTX I levels of rats were determined and the result indicated that compared with OVX model group the level of CTX I was largely decreased in rats that administrated with SZW 0.8 and 1.6 g/kg (Figure 3J). These findings indicated that SZW treatment attenuated osteoclast-mediated bone resorption.

### SZW promoted apoptosis of mature osteoclasts

**FIGURE 4:**
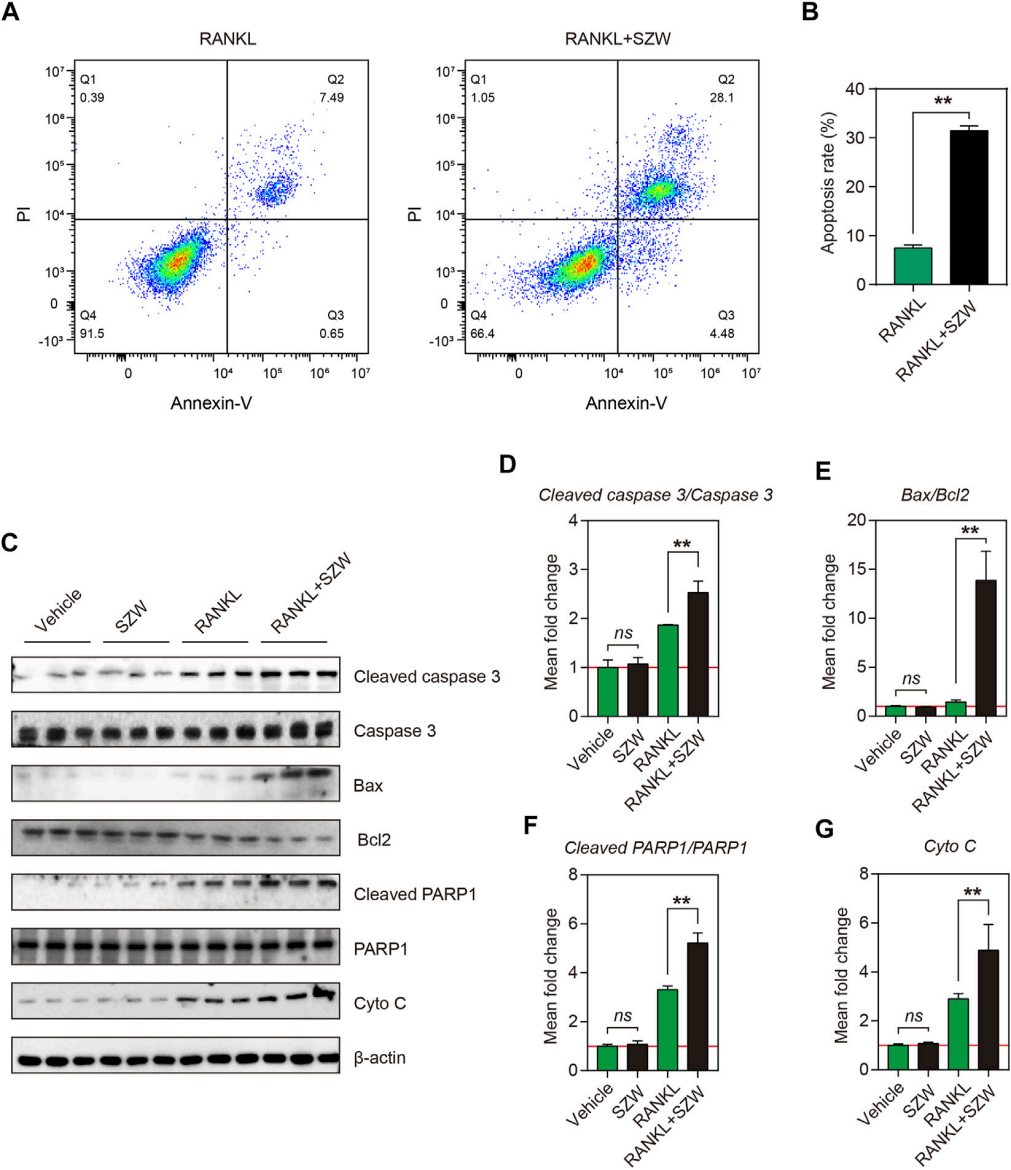
SZW promoted apoptosis of mature osteoclasts. RAW264.7 cells were treated with vehicle (H_2_O) or SZW in the presence or absence of RANKL **(A)** Representative flow cytometry pictures of osteoclast apoptosis **(B)** Quantitative statistics of apoptosis rate **(C)** Representative western blotting images showing Cleaved caspase 3, Caspase 3, Bax, Bcl2, Cleaved PARP1, PARP1, and Cytochrome c expression level in RAW264.7 **(D)** The mean fold change of Cleaved caspase 3/Caspase three ratio **(E)** The mean fold change of Bax/Bcl2 ratio **(F)** The mean fold change of Cleaved PARP1/PARP1 ratio **(G)** The mean fold change of Cytochrome c level. Data are expressed as the mean ± SEM (n = 3). ∗*p* < 0.05, ∗∗*p* < 0.01.

Apoptosis of osteoclasts is crucial in maintaining the osteoclast population in the bone’s basic multicellular unit (BMU). Apoptosis of osteoclasts in the osteoporotic bone is lower than in the osteoarthritic bone. The pro-apoptosis effect of SZW on mature osteoclasts was explored by inducing differentiation of RAW264.7 cells into mature osteoclasts using RANKL, followed treatment with vehicle or SZW for 24 h. To verify the effect of SZW on osteoclast apoptosis *in vitro*, treated osteoclasts were stained with Annexin V/PI and then detected by flow cytometry. After 24 h of SZW treatment, the osteoclasts underwent obvious apoptosis, i.e., there was a significant increase in the apoptosis rate (Figures 4A,B). Western blotting was performed to determine the expression of apoptosis-related proteins in treated mature osteoclasts, including cleaved caspase3, caspase3, Bax, Bcl2, cleaved PARP1, PARP1, and cytochrome c. Results showed that SZW treatment had no effect on the expression of cleaved caspase3/caspase3, Bax/Bcl2, cleaved PARP1/PARP1, and cytochrome C in RAW264.7 cells (Figures 4C–G). However, RANKL treatment increased the expression of these proteins in mature osteoclasts. Interestingly, treatment with SZW and RANKL further promoted the expression of cleaved caspase3, Bax, cleaved PARP1, and cytochrome c but inhibited that of Bcl2 (Figures 4C–G). Collectively, these results indicated that SZW increased the expression of apoptosis-related proteins in mature osteoclasts. Thus, SZW can alleviate osteoporosis by regulating the proliferation of osteoclasts.

### SZW suppressed NF-κB pathway activation *in vivo* and *in vitro*

**FIGURE 5:**
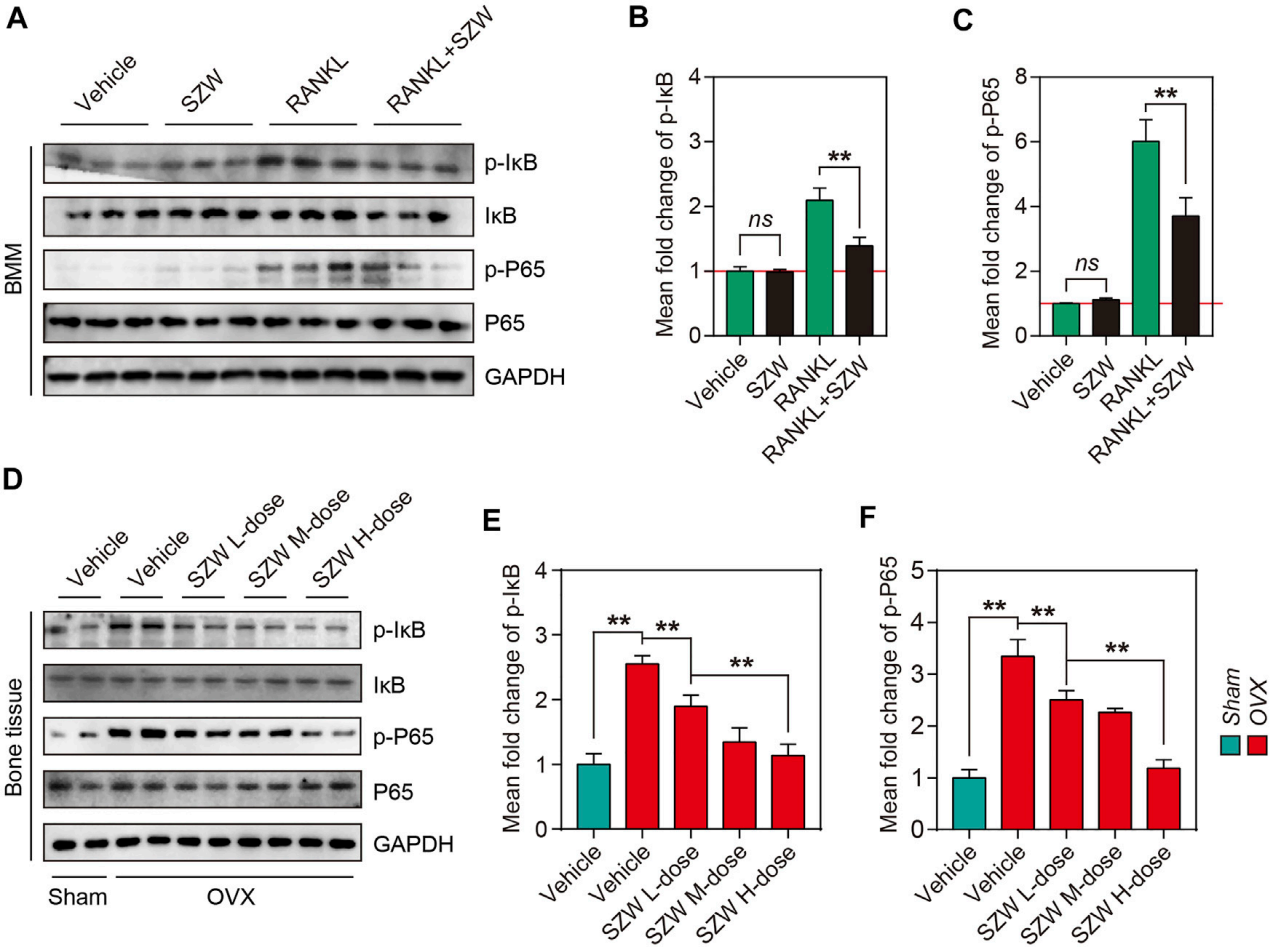
SZW suppressed NF-κB pathway activation *in vivo* and *in vitro.* Primary BMM cells were isolated from bone tissue of C57BL/6 mice, and BMMs were induced to differentiate into osteoclast precursors using M-CSF, and then treated with the indicated treatments **(A)** Representative western blotting images showing p-IκB, IκB, p-P65, and P65 expression levels in BMMs **(B)** The mean fold change of p-IκB/IκB ratio **(C)** The mean fold change of p-P65/P65 ratio **(D)** Representative western blotting images showing p-IκB, IκB, p-P65, and P65 expression in bone tissue of rats **(E)** The mean fold change of p-IκB/IκB ratio in rats **(F)** The mean fold change of p-P65/P65 ratio in rats. Data are expressed as the mean ± SEM (n = 3). ∗*p* < 0.05, ∗∗*p* < 0.01.

To further investigate the mechanism by which SZW exerts its inhibitory effect on osteoclastogenesis and pro-apoptosis effect on mature osteoclasts, we explored the effect of SZW on the NF-κB pathway. RANKL treatment for 30 min enhanced the phosphorylation of members of the NF-κB family, including IκB and P65. However, SZW treatment reversed this phenomenon (Figures 5A-C). Western blotting further confirmed that SZW treatment inhibited OVX-induced phosphorylation of IκB and P65 in a dose-dependent manner (Figures 5D-F). Collectively, the above findings indicate that SZW represses the NF-κB pathway activation, thus affecting the downstream signaling and transcription pathways.

## Discussion

In this study, we report for the first time the anti-osteoporosis properties of SZW *in vivo* and *in vitro*. Analysis of the uterine index, micro-CT parameters, and other pathological features in OVX rats revealed that SZW inhibited OVX-induced bone loss *in vivo*. Also, SZW suppressed osteoclastogenesis and osteoclast-induced bone resorption *in vivo* and *in vitro.* Mechanically, SZW regulates osteoclast activation by targeting the NF-κB pathway and promoting the apoptosis of mature osteoclasts to maintain osteoclast/osteoblast balance.

Bone remodeling depends on the balance between osteoclasts involved in bone resorption and osteoblasts involved in bone formation. (Chen et al., 2018; Kim et al., 2020). Osteoclasts, which regulate bone remodeling and promote excessive bone resorption, are the main cause of osteoclast-related bone disease. Therefore, inhibiting the excessive generation and improving the function of osteoclasts is an effective strategy to prevent and treat osteoclast-related bone diseases such as osteoporosis. Animal models for postmenopausal osteoporosis in mice, rats, and non-human primates are mainly induced through ovariectomy (Komori, 2015). In rats, bone loss after ovariectomy occurs in the proximal tibial metaphysis after 14 days, after 30 days in the femoral neck, and after 60 days in the lumbar vertebral body (Komori, 2015). Thus, the OVX-rat model is widely used for osteoporosis pathogenesis and pharmacology research. We observed significant bone loss in the rat femur 8 weeks after OVX (Figure 1). SZW treatment for 8 weeks alleviated OVX-induced bone loss in a dose-dependent manner (Figure 1), demonstrating the anti-osteoporosis potential of SZW. However, the specific effect of SZW on osteoclasts need to be further investigated using appropriate cell models.

M-CSF and RANKL treatment induces differentiation of mononuclear/macrophage lineage of hematopoietic cells in the bone marrow to osteoclasts (Takayanagi, 2021). RANKL induces differentiation of osteoclast precursor cells to multinuclear bone-resorbing cells via the RANK receptor. *In vitro* studies show that stimulation with M-CSF and RANKL induces the differentiation of osteoclast precursors, including BMMs, splenocytes, and peripheral blood monocytes, into osteoclasts (Kong et al., 2019; Honma et al., 2021). The RAW264.7 cells are also monocyte/macrophage-like cell lineage originating from the Abelson leukaemia virus-transformed BALB/c mouse lineage. *In vitro* models show that RANKL induces the differentiation of RAW264.7 cells into multinucleated cells, exhibiting the hallmarks of fully differentiated osteoclasts (Kong et al., 2019; He and Ma, 2020). In this study, the anti-osteoclastogenesis effect of SZW *in vitro* was evaluated using RAW264.7 cells. Upon RANKL stimulation, the cells differentiated into huge, multinucleated osteoclasts overexpressing TRAP, C-FOS, and NFATc1 proteins. These proteins, specifically expressed in osteoclasts, were also overexpressed in the bone tissues of OVX rats, indicating excessive osteoclastogenesis in the OVX-induced osteoporosis model. However, 25 and 50 μg/ml of SZW inhibited RANKL‐induced osteoclastogenesis *in vitro* with no toxic effects on the RAW264.7 cells. TRAP staining and Western blotting results revealed the anti-osteoclastogenesis effect of SZW in OVX rats *in vivo* (Figure 2). In addition, both *in vivo* and *in vitro* studies showed that SZW administration inhibited the expression of RANKL and OVX-induced osteolysis-related genes and proteins (Figure 3).

Bone metabolism is characterized by bone remodeling, continuous absorption, and the formation of new bones (Arias et al., 2018). The basic multicellular unit (BMU) responsible for bone resorption comprises, osteoclasts, osteoblasts, osteocytes, and lining cells (Jilka, 2003). A BMU has a lifespan of several months, while the lifespan of osteoclasts is about half a month (Sims and Martin, 2014). Apoptosis of osteoclasts is essential in maintaining the osteoclast population, the extent of bone resorption by the BMU, and the rate of BMU renewal. The apoptosis rate of osteoclasts is significantly lower in patients with osteoporosis than in healthy people (Soysa and Alles, 2019). Various signals and molecules that activate osteoclasts, especially M-CSF, inhibit osteoclast apoptosis (Soysa and Alles, 2019). M-CSF prevents osteoclast apoptosis through several mechanisms, including increasing Bcl-XL expression, which inhibits the cleavage of procaspase-9. This initiates apoptosis by activating microphthalmia-associated transcription factor (MITF), increasing Bcl-2 expression, and suppressing the expression of caspases three and 9. In this study, *in vitro* experiments revealed that SZW promotes apoptosis of mature osteoclasts by increasing the expression of pro-apoptosis proteins (Cleaved caspase 3, Cleaved PARP-1, Bax, and Cytochrome c) while down-regulating the expression of anti-apoptosis protein (Bcl2). However, SZW had no effect on the uninduced monocyte-macrophages (Figure 4).

RANKL and RANK binding activates several intracellular signaling pathways in osteoclast precursors, mainly including NF-κB and MAPKs (Amarasekara et al., 2018; An et al., 2019; Liu et al., 2020). NF-κB is essential for RANKL-induced osteoclastogenesis. The activation of c-Fos and NFATc1 is a downstream event in the NF-κB signaling pathway and is critical in the early phase of osteoclast development. Following stimulation with RANKL, phosphorylated IκBα is degraded to release p65, which is translocated into the nucleus to initiate the transcription of related genes. Herein, we demonstrate that SZW inhibits the NF‐κB signaling pathway by modulating the phosphorylation of IκB and P65 in BMMs, consistent with *in vivo* results. These findings suggest that SZW inhibits RANKL‐mediated osteoclast differentiation and the NF‐κB signaling pathway is a potential target for osteoclastogenesis treatment (Figure 5).

**FIGURE 6:**
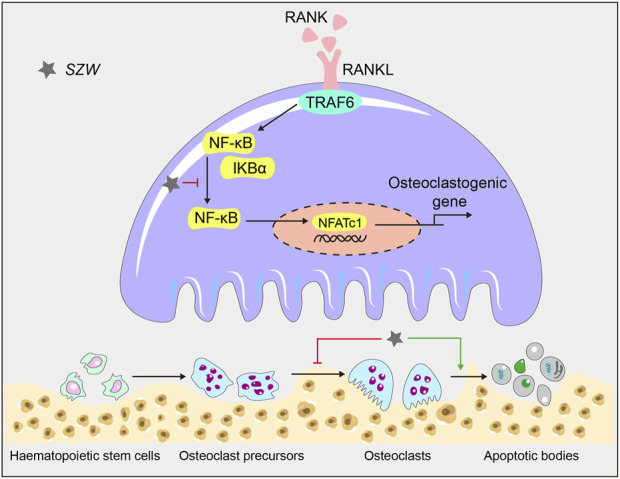
A schematic diagram illustrating the mechanism by which SZW inhibits osteoclastogenesis and promotes apoptosis in mature osteoclasts.

In conclusion, we demonstrated the anti-osteoporotic effect of SZW *in vivo* and *in vitro.* Specifically, we found that SZW suppressed osteoporosis by inhibiting osteoclastogenesis and promoting osteoclast apoptosis via the NF‐κB signaling pathways. Thus, SZW is potentially an effective candidate for osteoporosis treatment (Figure 6).

## Data Availability

The original contributions presented in the study are included in the article/Supplementary Material, further inquiries can be directed to the corresponding author.

## Abbreviations

OVX, ovariectomy; H&E, hematoxylin and eosin; RANKL, receptor activator of nuclear factor-κB ligand; TRAP, tartrate-resistant acid phosphatase, HRP, hormone replacement therapy; M-CSF, macrophage-colony stimulating factor; MAPK, mitogen-activated protein kinases; NFATc1, nuclear factor of activated T cells type c1; DMEM, Dulbecco’s Modified Eagle Medium; FBS, fetal bovine serum; BMMs, bone-marrow-derived monocytes/macrophages; CTX1, cross-linked C-telopeptide of type I collagen; MMPs, matrix metalloproteinases; BMD, bone mineral density; BV/TV, bone volume per tissue volume; Conn.D, connectivity densit; Tb.N, trabecular number; Tb.Sp, trabecular separation; Tb.Th, trabecular thickness.
