# Supplementary material for: Traditional Chinese decoction Si Zhi Wan attenuates ovariectomy (OVX)-induced bone loss by inhibiting osteoclastogenesis and promoting apoptosis of mature osteoclasts
Source: Front Pharmacol. 2022 Sep 13;13:983884. doi: 10.3389/fphar.2022.983884 (PMC9513524; doi:10.3389/fphar.2022.983884)
Supplement: Supplementary file 1 [file DataSheet1.PDF]

Supplementary Fig. 1

A

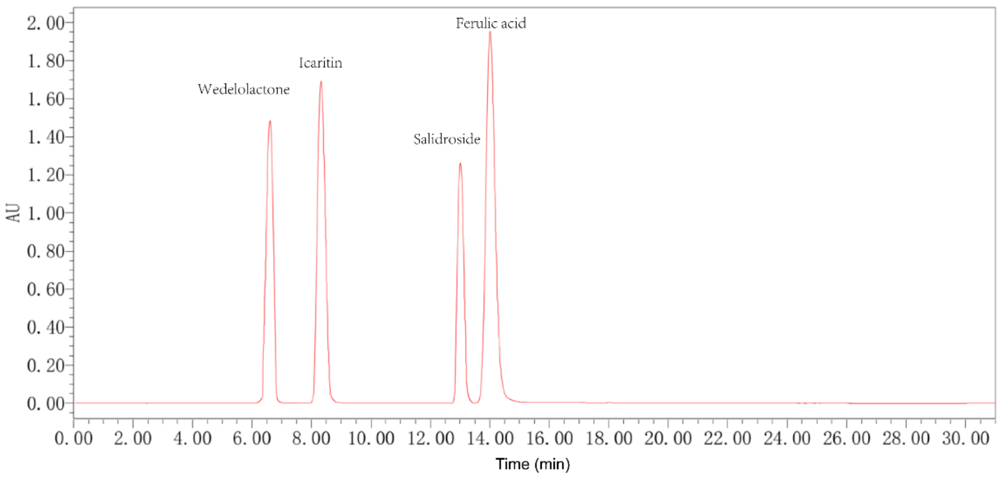

B

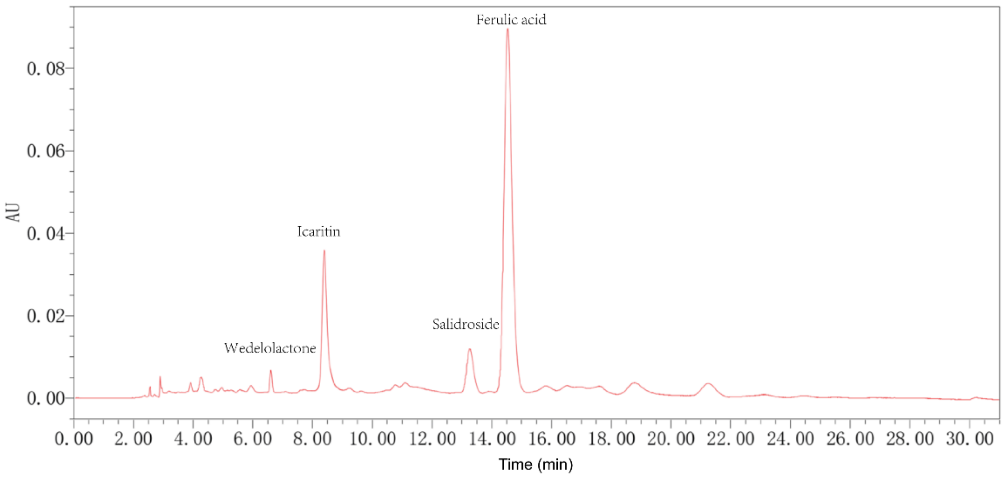

Supplementary Fig. 1. (A) HPLC chromatogram of reference compounds. (B) HPLC chromatogram of SZW extract.

Supplementary Fig. 2

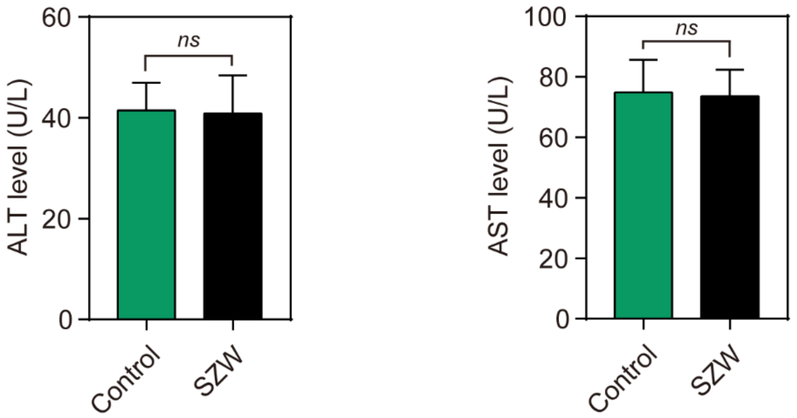

Supplementary Fig. 2. Effects of SZW on liver function in rats. After treated with SZW for 2 weeks, the serum of the rats in the control and SZW group was collected, then the ALT and AST levels of the rats were measured by an automatic biochemical analyzer.

Supplementary Table 1. Antibodies used

Antibody	Catalog	Company	Application	Dilution
TRAP	ab191406	Abcam	WB	1:1000
NFATC1	66963-1-Ig	Proteintech	WB	1:1000
c-Fos	AF0132	Affinity	WB	1:1000
MMP9	AF5228	Affinity	WB	1:1000
CTSK	DF6614	Affinity	WB	1:1000
Caspase 3/p17/p19	19677-1-AP	Proteintech	WB	1:1000
Bax	60267-1-Ig	Proteintech	WB	1:1000
BCL2	26593-1-AP	Proteintech	WB	1:1000
PARP1	13371-1-AP	Proteintech	WB	1:2000
Cytochrome c	10993-1-AP	Proteintech	WB	1:1000
Phospho-IKB alpha	AF2002	Affinity	WB	1:500
Phospho-NF-kB p65	AF2006	Affinity	WB	1:500
NF-kB p65	AF5006	Affinity	WB	1:500
IKB alpha	AF5002	Affinity	WB	1:500

Supplementary Table 2. Primers used for QPCR

Gene name	Forward	Reverse
ACP5	TGTGGCCATCTTTATGCT	GTCATTTCTTTGGGGCTT
NFATc1	CAACGCCCTGACCACCGATAG	GGCTGCCTTCCGTCTCATAGT
CTSK	GGGAGAAAAACCTGAAGC	ATTCTGGGGACTCAGAGC
c-Fos	AGGCCCAGTGGCTCAGAGA	GCTCCCAGTCTGCTGCATAGA
MMP9	AGTTTGGTGTCGCGGAGCAC	TACATGAGCGCTTCCGGCAC
